# *HIVEP3* cooperates with ferroptosis gene signatures to confer adverse prognosis in acute myeloid leukemia

**DOI:** 10.1002/cam4.4806

**Published:** 2022-05-10

**Authors:** Xiaoning Zhang, Xiaoyu Zhang, Kuo Liu, Wenwen Li, Jiazheng Wang, Peng Liu, Wanshan Ma

**Affiliations:** ^1^ Department of Clinical Laboratory Medicine The First Affiliated Hospital of Shandong First Medical University & Shandong Provincial Qianfoshan Hospital, Shandong Medicine and Health Key Laboratory of Laboratory Medicine Jinan PR China; ^2^ Department of Nephrotic The Fifth People's Hospital of Jinan Jinan PR China

**Keywords:** acute myeloid leukemia, bioinformatics analysis, ferroptosis, prognosis, the human immunodeficiency virus type I enhancer binding protein 3 gene

## Abstract

**Background:**

The human immunodeficiency virus type I enhancer binding protein (*HIVEP*) family, which contains zinc finger and acid‐rich (ZAS) domains, has been demonstrated to be implicated in vital biological processes, such as cell survival, tumor necrosis factor (TNF) signaling, and tumor formation. However, its expression patterns, prognostic relevance, and functional implications in acute myeloid leukemia (AML) remain elusive.

**Methods:**

We inspected *HIVEP* mRNA expression levels in datasets from The Cancer Genome Atlas (TCGA) and GSE24006. Survival analyses were orchestrated using the web‐based bioinformatics platforms and R studio in two AML cohorts. Prognostic value and capacity were assessed by Cox regression analyses. Association of *HIVEP3* expression levels with clinical characteristics were analyzed with R and UALCAN. Subsequentially, functional enrichment analyses were operated to interpret *HIVEP3* co‐expressed gene clusters. A prognostic gene signature was created by the least absolute shrinkage and selection operator (LASSO) regression algorithm. Moreover, bone marrow aspirate smears of AML patients were stained for *HIVEP3* by immunohistochemistry (IHC). *HIVEP3* expression was examined by qRT‐PCR in leukemia cell lines treated with ferroptosis compounds in vitro.

**Results:**

Augmented transcriptional levels of *HIVEP2* and *3* were noted in AML patients (*p*<0.001). *HIVEP3* not only could confer adverse prognosis independently in AML patients, but also was associated with AML subtypes, age, cytogenetic risk, and disease‐related molecules. Co‐expressed gene clusters of *HIVEP3* were enriched in functional pathways related to AML leukemogenesis, such as ribosome, metabolism, and calcium signaling. Combined with multiple tumorigenesis signaling pathways, we proposed an integrated LASSO model with *HIVEP3* and ferroptosis regulators *AIFM2* and *LPCAT3*, to predict the outcome for AML patients. Furthernore, altered *HIVEP3* expression at the mRNA or protein level was confirmed in sorted leukemia cells and blast cells in bone marrow tissues. In vitro experiments authenticated the involvement of *HIVEP3* in ferroptosis signaling pathways.

**Conclusions:**

Our findings suggest that *HIVEP3* is a de novo independent prognostic indicator, and the crosstalk between *HIVEP3* and ferroptosis signaling pathways may inspire a specific perspective on the oncological network of AML.

## Graphical abstract

The aberrant expression pattern and prognostic implications of *HIVEP3* in AML were explored and validated based on versatile bioinformatics tools. A LASSO model which combined *HIVEP3* with ferroptosis regulators was created to predict the prognosis for AML patients.

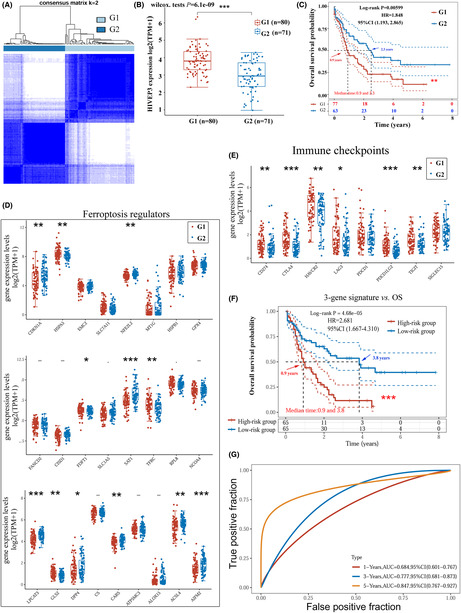

## 1 INTRODUCTION

Acute myeloid leukemia (AML) is a severe myeloproliferative disorder featured by unlimited expansion of blasts cells. World Health Organization (WHO) classification and diagnostic criteria (2016 edition) for myeloid neoplasms and acute leukemia emphasize the prognostic relevance of cytogenetics, and molecular genetics, in addition to common clinical, morphologic, and immunophenotypic entities, due to the genetic complexity of the disease.^1^, ^2^ Thence clinical outcomes of AML patients are estimated by age, cytogenetic abnormalities, and specific gene expressions. A batch of recurring chromosomal and genetic lesions, such as *PML‐RARα*, mutations of *FLT3*, *TP53*, *DNMT3A*, *RUNX1*, and *NPM1*, related to disruptions of oncogenes and tumor suppressor genes were recommended. The current risk and prognosis prediction strategy based on age and gene mutations are not adequate or effective enough since the therapeutic landscape of AML has changed over the past 10 years. Many endeavors have been made to ascertain de novo biomarkers and improve risk stratification and prognostic assessment in different AML subgroups with the assistance of next‐generation sequencing (NGS) technology. Gene signatures such as *HMGA2*,^3^ *FHL1*, *HOPX*, and *FAM124B* ^4^ have been designated as effective prognosis indicators. Although their clinical practice is limited, the risk and prognosis assessment system was compensated for to some extent. Besides, the potential NGS implementation for genetic diagnosis and prognosis has been explored by data interpretation between NGS data and clinical characteristics. To get a more comprehensive understanding of the clinical performance and biological process of AML, we screened prognosis‐related genes in AML patients and observed aberrant expression and prognostic value of *HIVEP3* via bioinformatic tools combined with wet‐lab experiments.

The *HIVEP* gene family consists of three members, *HIVEP1*, *2*, and *3*, which encodes a huge protein harboring four to five zinc fingers. *HIVEP1* and *2* are expressed ubiquitously, but *HIVEP3* expression is only detected in lymphoid and neural tissues.^5^, ^6^ All three genes are supposed to perform multifaceted functions due to the long chains and abundant functional folding units. Hivep proteins could bind to DNA targets, but also interact with various transcription or signaling transduction molecules. Hivep3, which possesses unique spatial and temporal expression patterns compared with Hivep1 or Hivep2, may exert more diverged functions.^7^ As a transcription factor, Hivep3 binds to the κB motif of genes such as *S100A4*, involved in cell progression and differentiation.^8^ Transfection of *S100A4* in leukemia cells inhibits p53 signaling, thence reduces apoptosis and increases proliferation in vitro.^9^ A high *S100A4* expression was associated with poor OS in AML.^10^ Hivep3 also interferes with nuclear factor *NF‐κB* and c‐Jun N‐terminal kinase/JNK‐mediated responses, including apoptosis and proinflammation.^11^ Experimental outcomes in mouse models with *HIVEP3* deficiency suggest *HIVEP3* is implicated in cell growth controlling and tumorigenesis. The research above gave clues that *HIVEP* genes could be involved in oncological signaling pathways such as apoptosis and immune microenvironment. Neither did previous studies refer to the definite expression status and functions of *HIVEP* genes in AML, nor does evidence exist that they underlie the interplay among oncological signaling pathways.

## 2 MATERIALS AND METHODS

### 2.1 Gene expression profiling

Transcriptional expression levels of *HIVEP1*, *2*, and *3* were inspected in pan‐cancer cohorts derived from TCGA databases, while those of normal tissue samples from the Genotype‐Tissue Expression (GTEx). The RNA‐seq data in TPM (transcripts per kilobase million reads) format were processed through Toil in UCSC XENA.^12^ A dataset at the NCBI Gene Expression Omnibus (GEO, accession GSE24006), which includes AML leukemic stem cells (LSC, CD34^+^CD38^−^; *n* = 7), AML leukemic progenitor cells (LPC, CD34^+^CD38^+^; *n* = 7), AML Blasts (CD34^−^; *n* = 7), and normal hematopoietic stem cells (HSC, CD34^+^; bone marrow and cord blood, *n* = 7), sorted from peripheral blood and/or bone marrow, was adopted to inspect the *HIVEP3* mRNA expression in normal and leukemic subpopulations. Raw CEL intensity data were normalized using MAS5 algorithm.^13^

### 2.2 Survival analysis

A TCGA‐LAML cohort containing 151 AML patients with high‐throughput sequencing (RNA‐Seq), mutation status, and detailed clinical information was obtained as previously described.^14^ The survival results were displayed by Kaplan–Meier (KM) curves.

### 2.3 Univariate and multivariate Cox regression analyses

As previously reported, univariate and multivariate Cox regression analyses were employed to screen for the potential prognostic indicators in the TCGA‐LAML cohort (*n* = 151).^14^ The forest plots with assorted variables were generated using “survival”, “survminer”, “forestplot” R packages. The age, cytogenetic risk, and gene mutations were treated as dichotomous or categorical variables, while the leukocyte count and blast cell percentage as continuous variables. The median expression level of each variable was adopted as the threshold value to dichotomize the cohort.

### 2.4 The LASSO Cox regression

The LASSO Cox regression was conducted in the TCGA‐LAML cohort to select a simple but powerful prognostic model from a combination of *HIVEP3*‐related ferroptosis regulators and immune checkpoints. The optimal penalty parameter λ correlated with the minimum10‐fold cross‐validation was selected via the R package “glmnet”(v 4.1–1). The polygenic risk scoring formula below was built to calculate the risk score of each patient: Risk score = ∑i=1nCoef×xii. Coef (coefficient) of selected feature is represented by lambda parameter. The TCGA‐LAML cohort was stratified into the high‐ and low‐risk score groups using the median risk score as the cutoff value.

### 2.5 Tissues and immunohistochemical staining

Twenty bone marrow samples of AML patients and 11 samples of healthy people were selected from archival paraffin wax‐embedded tissue blocks in the pathology department. Informed consent was obtained from all patients before the study. This research was done with the approval of the Medical Ethics Committee of Shandong First Medical University according to the Declaration of Helsinki. Immunohistochemical staining was performed according to standard protocols using *HIVEP3* antibody (A20298, 1:100; Abclonal). Briefly, the tissue slides were deparaffinized, retrieved with citrate buffer, and incubated with primary antibody for 60 min, then anti‐rabbit secondary antibody for 60 min, at room temperature. Subsequently, the slides were incubated in DAB (AR1021, Boster), counterstained with hematoxylin, and mounted. Three pathologists evaluated the *HIVEP3* staining under an optical microscope (ZEISS), ranging from negative, slight, moderate, or severe. The slides were imaged using the ZEISS Axio observer.

### 2.6 Cell culture and treatment

Two AML cell lines THP‐1 and KG‐1 were purchased from American Type Culture Collection (ATCC) and cultured in RPMI 1640 (Gibco) or IMDM medium (Macgene) supplemented with 10% fetal bovine serum (TBD, Tianjin) and 100 units/ml of penicillin–streptomycin (Beyotime, Shanghai) in a humidified atmosphere with 5% CO_2_ at 37 °C. The cells were seeded in six‐well plates at 2.0 × 10^5^/ml. AML cells were treated with 50 μM Erastin (Er, MCE, Shanghai), 1 μM ferrostatin‐1 (Fer‐1, MCE), and dimethyl sulfoxide (DMSO, Beyotime) for 24 h, respectively. Subsequently, cells were harvested with TRIzol reagent (TIANGEN).

### 2.7 Quantitative real‐time PCR (qRT‐PCR)

Total RNA in cells was extracted using the TRIzol method. The ReverTra Ace qPCR RT kit (Toyobo, DYF/FSQ‐101) was used to synthesized complementary DNA (cDNA). *HIVEP3* and an internal control β‐actin were amplified using SYBR Green Real‐time PCR Master Mix (Toyobo, DYF/QPK‐201) on ABI QuantStudio 5 (Applied Biosystems). The primer sequences were as follows: *HIVEP3* _(119 bp) forward 5’‐ATCGAAGCATCCGTCCACATC ‐3′ and reverse 5’‐ATGGGGTCAACCAGTTGCC ‐3′; β‐actin_(180 bp) forward 5′‐ CTCACGAAACTGGAATAAGC ‐3′ and reverse 5′‐ AAGCCACACGTACTAAAGGT −3′. Relative quantification of the mRNA levels was calculated using the 2^−△△Ct^ method.

### 2.8 Statistical analysis

Log2 (TPM + 1) values from TCGA‐LAML cohort were recognized as non‐normally distributed data by Shapiro–Wilk normality test, which were described as medians and interquartile range (IQR) and examined by the Mann–Whitney *U* test. The dot plots were drawn through “ggplot2” package in R studio (3.6.3).

The TCGA‐LAML cohort (*n* = 151) was dichotomized into high‐ and low‐expression groups using the median *HIVEP3* expression level as a cutoff. The *p*‐values and the hazard ratios (HR) with 95% confidence interval (CI) in survival analyses were generated from univariate Cox proportional hazard regression and log‐rank test. Time‐dependent receiver operating characteristic (ROC) analyses were employed to quantify the predictive accuracy of a single gene or a conceptual gene set. Methods above have been implemented in R packages “survival”, “survminer”, “pROC”, and “ggplot2”.

Distribution patterns related to *HIVEP3* expression level in AML subgroups with different clinical features or genetic abnormalities were examined by chi‐squared tests or Fisher's exact tests as appropriate using R software.

Web‐based bioinformatics tools such as GEPIA2,^15^ PrognoScan,^16^ Linkedomics,^17^ UALCAN,^18^ and WebGestalt^19^ were utilized to perform survival analysis, correlation analysis, and functional enrichment analysis (supplementary).

The R software package “ConsensusClusterPlus” was applied for consistency analysis. The number of clusters are set at 2, and 80% of the total objects are drawn 100 times. Gene signatures of fatal pathways such as ferroptosis, N6‐methyladenosine (m6A), and immune microenvironment are derived from published literature.^20^, ^21^, ^22^, ^23^ The heatmap reserves genes with SD >0.1.

Analyses between the two groups of normally distributed qRT‐PCR data were performed using Student's *t*‐tests. Graphpad Prism 8.0.2 software was applied for statistical analyses and visualization. The level of significance was *p*‐value < 0.05.

## 3 RESULTS

### 3.1 Augmented expression levels of *HIVEP* gene family in AML

**FIGURE 1:**
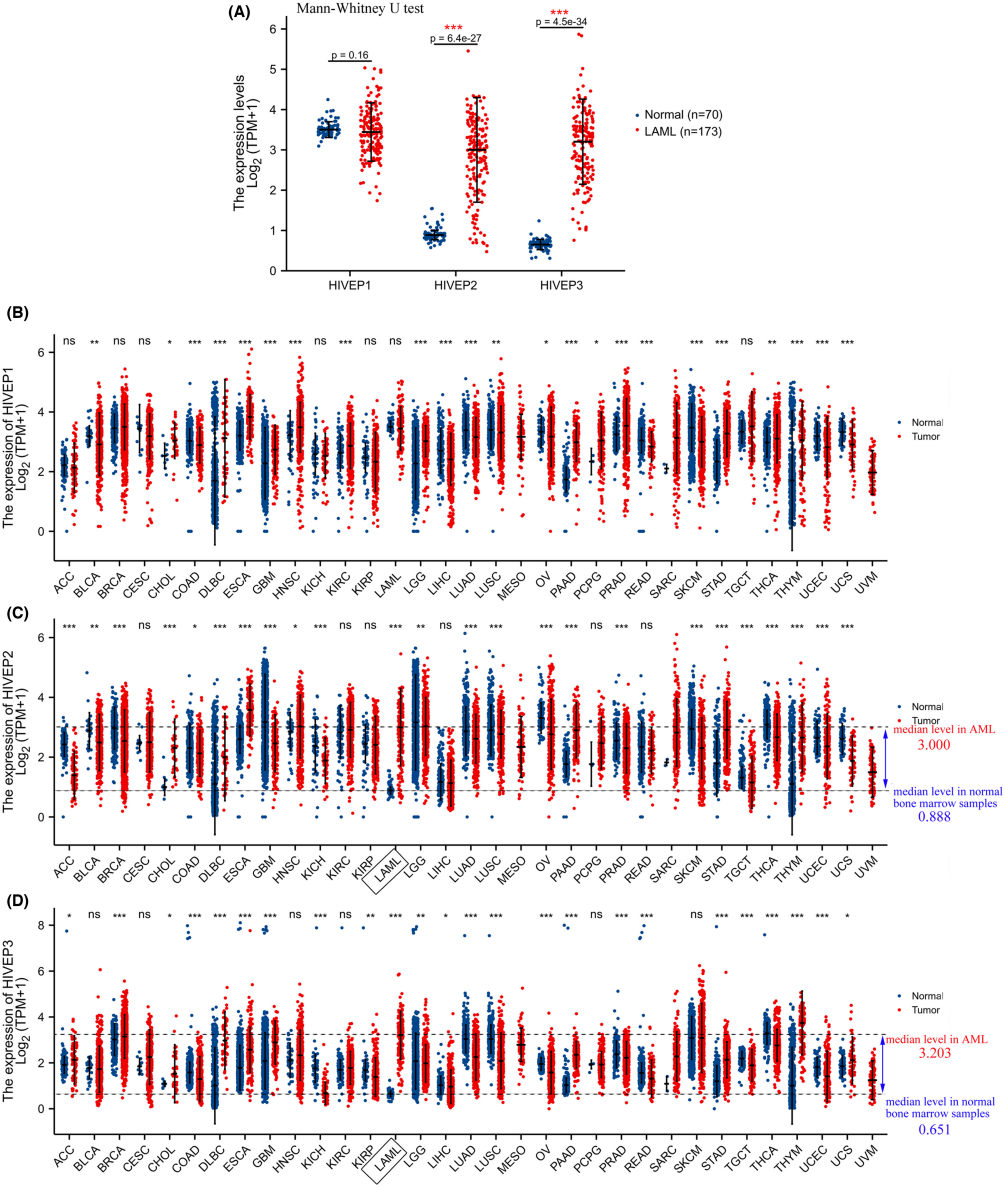
*HIVEP* mRNA expression profiles in pan‐cancer. (A) Dot plots show the expression profiles of *HIVEP1*, *HIVEP2*, and *HIVEP3* in bone marrow samples of patients in the TCGA‐AML cohort (*n* = 173) compared to those in normal samples (*n* = 70) from GTEx. Expression patterns of (B) *HIVEP1*, (C) *HIVEP2*, and (D) *HIVEP3* were profiled in pan‐cancer. Expression analysis was conducted in RNA‐Seq datasets by R studio. The transcriptional levels were log‐transformed with the formula log_2_ (TPM + 1). A Mann–Whitney *U* test was used to evaluate the differences of expression levels between tumor and histologically healthy tissues with error bars designating median ± IQR. Gray dot lines indicate median mRNA levels. Asterisks denote different *p* values (*p < 0.05, **p < 0.01, ***p < 0.001). TPM, transcripts per kilobase million reads. IQR, interquartile range. ns, not significant

The transcription levels of *HIVEP* genes in the bone marrow of 173 TCGA‐LAML patients and 70 GTEx normal tissues were assembled, profiled, and visualized by dot plots via R studio (Figure 1A). Gene expression analyses illustrated that the transcriptional levels of *HIVEP2* and *HIVEP3* were conspicuously increased (*p* < 0.001) in AML samples, while that of *HIVEP1* showed no difference.

We also profiled the expression levels of *HIVEP* genes in pan‐cancer. The dot plots indicated a wide disparity among *HIVEP* genes expression levels in different cancer types (Figure 1B–D). We assumed that *HIVEP* genes are expressed in an organ‐specific pattern during the developmental process, among which *HIVEP2* and *3* exhibit the most divergent expression status between AML samples and normal tissues (3.000 ± 1.298 vs. 0.888 ± 0.116 for *HIVEP2*, *p* < 0.001; 3.203 ± 1.056 vs. 0.651 ± 0.126 for *HIVEP3*, *p* < 0.001). However, to what extent such bone marrow‐specific expression patterns in AML may contribute to myeloid leukemogenesis and facilitate the prognosis prediction remains unclear.

### 3.2 Prognostic implications of *HIVEP2* and *3* in AML

**FIGURE 2:**
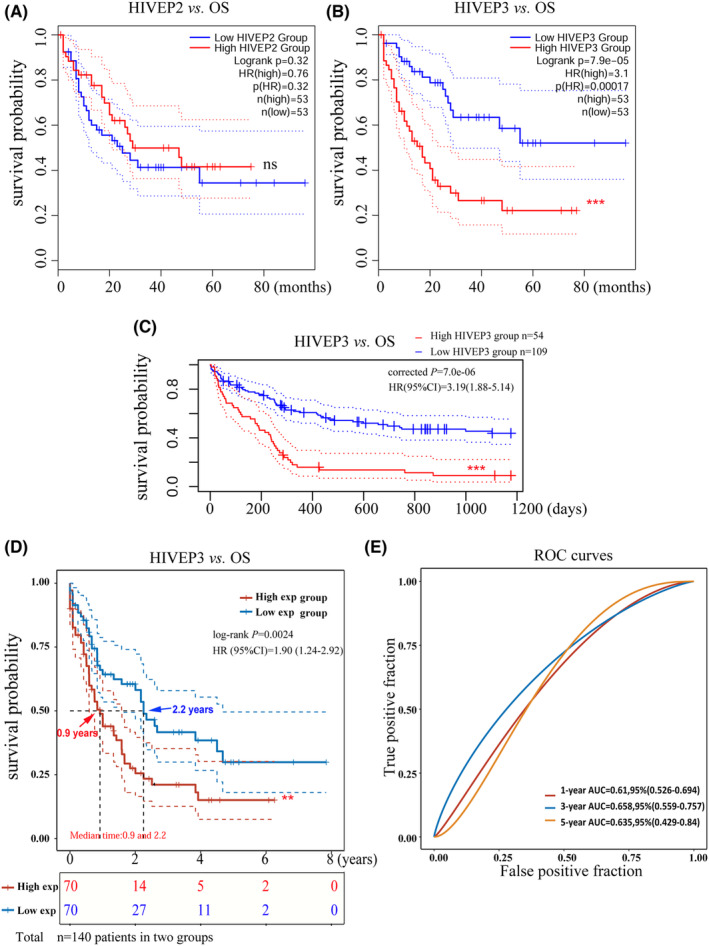
Prognostic implications of *HIVEP2* and *3* in AML patients. (A,B) Prognosis analyses were conducted based on the mRNA expression levels and survival status in the TCGA‐LAML cohort (*n* = 106 after case‐wise deletion) via GEPIA2. The prognostic value of *HIVEP3* was validated through (C) PrognoScan database in AML CG (1999–2003) cohort (*n* = 163, GSE12417‐GPL97) and (D) R studio in the TCGA‐LAML cohort (*n* = 140). *p*‐values and HRs were computed by log‐rank test and Cox regression to draw Kaplan–Meier (KM) curves. 95%CI was denoted as dotted lines. Patients were dichotomized in GEPIA2 and R into a high‐ (solid red line) and a low‐ expression group (solid blue line), with the median mRNA expression level as the cutoff value, while the cut point was set at 0.67 in PrognoScan based on continuous gene expression measurement. The median survival time was indicated as red and blue arrows, respectively. (E) The predictive accuracy of *HIVEP3* was assessed by time‐dependent ROC analysis at different time points. AUC values represent the prediction ability. AUC, area under the curve; CI, confidence interval; HR, hazard ratio; OS, overall survival; ROC, receiver operating characteristic

Survival analysis was performed through the GEPIA2 (Figure 2A,B) and PrognoScan (Figure 2C) databases via different data processing algorithms in the AML cohorts. Intriguingly, only *HIVEP3* aberration was associated with poor overall survival (OS) (HR [high] = 3.1; log‐rank *p* = 7.9e‐05), while *HIVEP2* showed no association with OS (Figure 2A,B). The prognostic significance of *HIVEP3* was confirmed through the PrognoScan pipeline in the cytogenetically normal AML cohort GSE12417 (*n* = 163, *p* = 7.0e‐06, Figure 2C). *HIVEP3* emerged as a risk predictor for OS with HR = 3.19 (95% CI, 1.88–5.14, Figure 2C).

We conducted univariate Cox proportional hazard regression with the gene expression data and survival status to screen for potential prognostic candidates in the TCGA‐LAML cohort (*n* = 151) via R studio. Among 754 prognosis‐related genes with a Cox *p*‐value < 0.001 (data not shown), enhanced expression of *HIVEP3* was remarkably associated with adverse OS (HR = 1.90, 95%CI [1.24–2.92]; Cox *p* = 0.0008). The prognostic evaluation capacity of *HIVEP3* was validated by KM survival analysis (log‐rank *p*‐value = 0.0024, Figure 2D). The median survival time of the *HIVEP3* high‐ and low‐expression groups are 0.9 years and 2.2 years, respectively. Time‐ROC analysis of *HIVEP3* was performed to determine the predictive accuracy. The area under the curve (AUC) was 1‐year AUC = 0.61, 3‐year AUC = 0.66, 5‐year AUC = 0.64, respectively (Figure 2E).

Some clinical features, including age, leukocyte count, blast cell proportion, and multiple cytogenetic mutations, would be likely to provoke an impact on the prognosis of AML. Genome‐wide prognosis analyses have been conducted to screen for prognosis‐related variables, by which the prognostic significance of *FHL1*, *HOPX*, *HMGA2*, and *FAM124B* was recently identified and reported.^3^, ^4^ Thence, we conducted Cox regression analyses to determine whether *HIVEP*3 was a powerful AML OS‐related gene.

**FIGURE 3:**
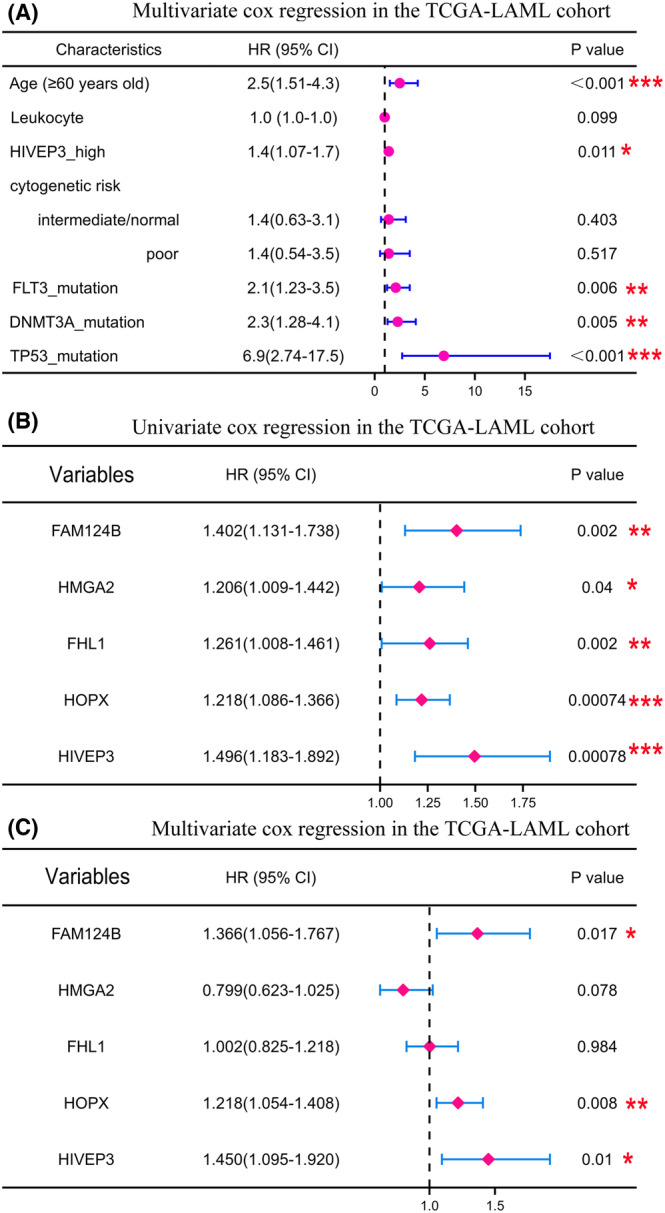
Prognostic capacity of *HIVEP3* among multiple variables related to OS in AML patients. (A) The forest plots were generated in R studio by multivariate Cox regression analyses with HR, 95%CI, and *p*‐values. (B) Univariate and (C) multivariate Cox regression analyses were visualized in the forest plots to show the prognostic significance of a set of individual genes

Clinicopathological characteristics, prognostic markers, and *HIVEP3* were selected for Cox regression in the TCGA‐LAML cohort (Figure S1 and Figure 3). The forest plots represented the prognostic significance of augmented *HIVEP3* expression for dismal outcomes of AML patients (HR = 1.4; 95% (CI), 1.07–1.7; *p* = 0.011), independent of age, poor cytogenetic risk, and specific gene mutations (Figure 3A and Figure S1). Among those known prognostic candidates, the comparably crucial prognostic value of *HIVEP3* was highlighted (*p*‐value = 0.010), with a higher HR (HR = 1.450, Figure 3B,C). The results above verified that *HIVEP3* is an efficient prognosis predictor independent of multiple disease‐related factors in AML patients.

### 3.3 Association of *HIVEP3* expression level with AML clinicopathological characteristics

Eight subtypes of AML (M0‐M7) were systematically categorized by the French‐American‐British (FAB) classification strategy with differing morphologic features and histochemical stains.^24^ Later on, WHO classification takes into account recurring chromosomal and genetic abnormalities.^25^ These cytogenetic abnormalities, combined with clinical characteristics, contribute to the current risk stratification for AML patients.

**FIGURE 4:**
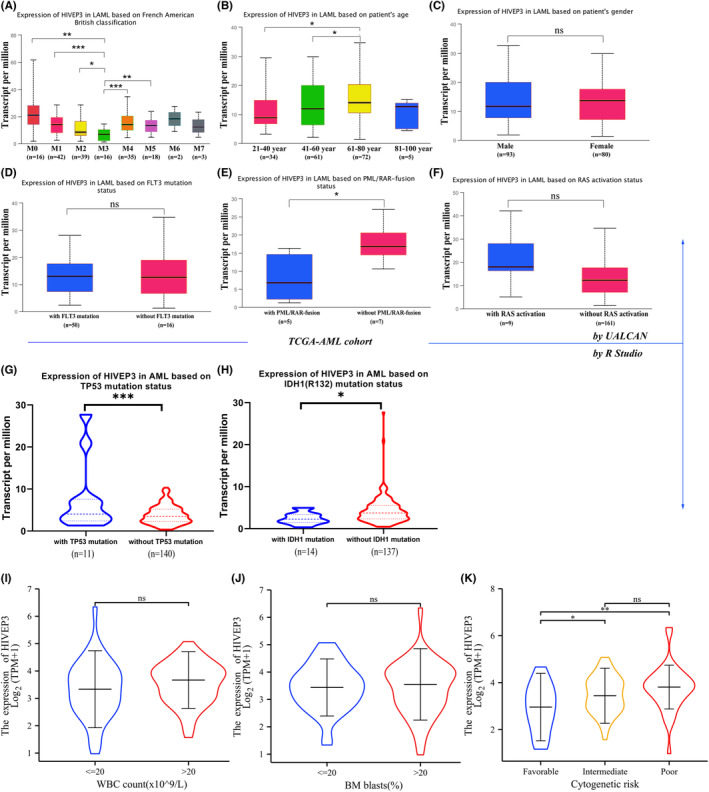
The mRNA expression levels of *HIVEP3* in AML subgroups (UALCAN). (A) *HIVEP3* TPM levels were displayed by box plots in different FAB subtypes. (B–F) TPM values of *HIVEP3* were used for comparison across AML subtypes based on (B) age, (C) gender, (D) *FLT3* mutation, (E) *PML/RAR*‐fusion, and (F) *RAS* activation status. (G–K) *HIVEP3* expression levels were compared by R studio in AML subtypes based on (G) *TP53* mutation, (H) *IDH* mutation, (I) peripheral blood WBC count, (J) bone marrow blasts percentage, and (K) cytogenetic risk

The distribution of *HIVEP3* expression was inspected in AML patients with multiple clinical and molecular characteristics. The elevated expression level of *HIVEP3* was associated with adverse (*p* = 0.009) and complex cytogenetics (*p* = 0.028). The *HIVEP3*‐high and‐low groups showed diverse distribution among the FAB subtypes (*p* = 0.024). Most of those with M3 showed lower *HIVEP3* expression (80.0%). There was no relation between *HIVEP3* expression and gender, WBC count, percentage of blast cells, or several specific gene mutations (Table 1). Next, *HIVEP3* expression levels were profiled in different AML subtypes through UALCAN and R studio (Figure 4). Consistently, *HIVEP3* expression was augmented in M0, M1, M2, M4, and M5 compared with that in M3 (Figure 4A). The expression level of *HIVEP3* was higher in the age 61–80‐year‐old group than that in younger age groups (Figure 4B). *HIVEP3* expression was elevated in samples without *PML‐RAR* fusion (*p* = 0.023, Figure 4E), with TP53 mutation (*p*<0.001, Figure 4G), without *IDH*‐R132 mutation (*p* = 0.042, Figure 4H), and samples with intermediate and poor cytogenetic risk (Figure 4K). *PML‐RAR* fusion and *IDH*‐R132 mutation are two common favorable prognosis indicators. Meanwhile, it shows no difference between subgroups with and without the other 22 gene mutations such as *RUNX1*, *DNMT3A*, *WT1*, and *CEBPA* ^*double*^ (data not shown).

**TABLE 1:** Association of *HIVEP3* expression levels with clinical and genetic characteristics. *p*‐values were created by chi‐squared test and ^△^ Fisher's exact test

Characteristics	Low expression of HVEP3	High expression of HIVEP3	*p‐value*
	*n* = 75	*n* = 76	
Age, *n* (%)			0.114
<=60	49 (32.5%)	39 (25.8%)	
>60	26 (17.2%)	37 (24.5%)	
Gender, *n* (%)			0.928
Female	33 (21.9%)	35 (23.2%)	
Male	42 (27.8%)	41 (27.2%)	
WBC count (x10^9^/L), *n* (%)			0.140
<=20	43 (28.7%)	34 (22.7%)	
>20	31 (20.7%)	42 (28%)	
BM blasts (%), *n* (%)			0.572
<=20	32 (21.2%)	28 (18.5%)	
>20	43 (28.5%)	48 (31.8%)	
PB blasts (%), *n* (%)			0.571
<=70	38 (25.2%)	34 (22.5%)	
>70	37 (24.5%)	42 (27.8%)	
Cytogenetic risk, *n* (%)			0.009**
Favorable	21 (14.1%)	10 (6.7%)	
Intermediate	42 (28.2%)	40 (26.8%)	
Poor	11 (7.4%)	25 (16.8%)	
FAB classifications, *n* (%)			0.028*
M0	5 (3.3%)	10 (6.7%)	
M1	17 (11.3%)	18 (12%)	
M2	24 (16%)	14 (9.3%)	
M3	12 (8%)	3 (2%)	
M4	11 (7.3%)	18 (12%)	
M5	4 (2.7%)	11 (7.3%)	
M6	1 (0.7%)	1 (0.7%)	
M7	1 (0.7%)	0 (0%)	
Cytogenetics, *n* (%)			0.024*
Normal	36 (26.7%)	33 (24.4%)	
+8	5 (3.7%)	3 (2.2%)	
del (5)	1 (0.7%)	0 (0%)	
del (7)	1 (0.7%)	5 (3.7%)	
inv (16)	3 (2.2%)	5 (3.7%)	
t(15;17)	8 (5.9%)	3 (2.2%)	
t(8;21)	7 (5.2%)	0 (0%)	
t(9;11)	0 (0%)	1 (0.7%)	
Complex	8 (5.9%)	16 (11.9%)	
*FLT3* mutation, *n* (%)			0.956
Negative	22 (15%)	23 (15.6%)	
Positive	52 (35.4%)	50 (34%)	
*IDH1 R132* mutation, *n* (%)			0.235
Negative	9 (6%)	4 (2.7%)	
Positive	65 (43.6%)	71 (47.7%)	
*IDH1 R140* mutation, *n* (%)			1.000
Negative	6 (4%)	6 (4%)	
Positive	69 (46.3%)	68 (45.6%)	
*IDH1 R172* mutation, *n* (%)			0.497^△^
Negative	2 (1.3%)	0 (0%)	
Positive	73 (49%)	74 (49.7%)	
*RAS* mutation, *n* (%)			0.276^△^
Negative	2 (1.3%)	6 (4%)	
Positive	73 (48.7%)	69 (46%)	
*NPM1* mutation, *n* (%)			0.693
Negative	18 (12%)	15 (10%)	
Positive	57 (38%)	60 (40%)	

* *p* < 0.05.

** *p* < 0.01.

Moreover, Pearson's correlation analyses were conducted between *HIVEP3* and common disease‐related genes (Figure S2). The statistical scatter plots displayed that the *HIVEP3* expression was positively associated with *FLT*, *HIF1A*, *FHL1*, and *RUNX3*. Meanwhile, a negative relationship was observed between *HIVEP3* and *MPO* and *VEGF* (Figure S2).

### 3.4 Co‐expressed genes of altered *HIVEP3* expression in AML

**FIGURE 5:**
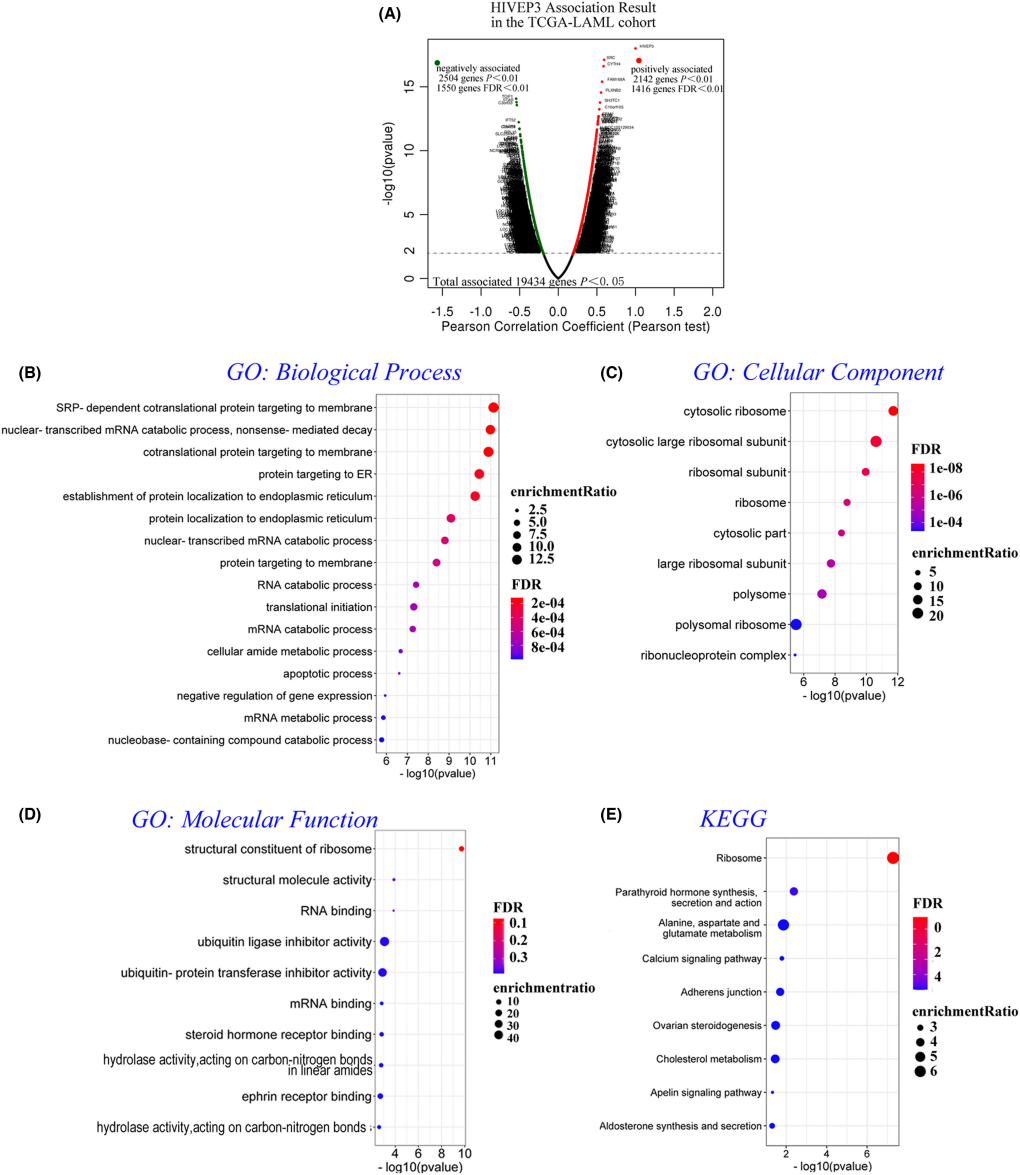
*HIVEP3* and co‐expressed genes were subjected to functional enrichment analyses in the TCGA‐AML cohort (WebGestalt). (A) The volcano plot of red and green dots assembles 19,434 *HIVEP3* associated gene clusters in the TCGA‐AML cohort by Pearson's test (*p* < 0.05). One thousand four hundred and sixteen positively associated genes are represented in the right sector, while 1550 negatively associated genes are in the left sector (FDR < 0.01). (B–D) GO of three aspects including (B) biological processes, (C) cellular components, and (D) molecular functions, and (E) KEGG analyses were annotated by bubble charts. Bubbles in graded colors and various sizes illustrate the FDR and enrichment ratio of representative enrichment categories and pathways. Thresholding criterion was set at (−log10) *p*‐value = 1.3. FDR, false discovery rate

To further explore whether *HIVEP3* was involved in leukemogenesis, we assayed mRNA sequencing data from 173 AML patients in TCGA. 19,434 genes associated with *HIVEP3* were screened out utilizing the LinkedOmics database, reflecting the considerable impact of the core gene *HIVEP3* on AML pathogenesis. Red dots represented the positively associated 1416 gene clusters, whereas 1550 green dots displayed the negatively associated gene clusters in the volcano plot (FDR < 0.01, Figure 5A).

Meanwhile, top20 co‐expressed genes were listed with Pearson's correlation (Table S1), which reflect changes in transcription initiation, cell adhesion, and ribosome/mitosis composition. We also evaluated the prognostic value of these co‐expressed genes in patients with AML by survival analysis. Eleven potential prognosis indicators in the positively associated gene set and 12 ones in the negatively associated gene set were filtrated. High transcriptional levels of *CYTH4*, *ITGAL*, and *NEK6* were significantly related to dismal OS in patients with AML. In contrast, genes such as *CALR*, *CASP6*, and *FXR1* displayed protective potential for OS (Table S1). The results were congruent with the correlation of *HIVEP3* overexpression with OS.

### 3.5 Gene ontology (GO) and Kyoto Encyclopedia of Genes and Genomes (KEGG) analyses of *HIVEP3* and co‐expressed genes in patients with AML

We further predicted the functions and pathways of *HIVEP3* and its top100 positively and negatively associated gene clusters through GO and KEGG analyses in TCGA‐LAML. The bubble diagrams displayed that the gene clusters were located in biological processes which regulate the mRNA catabolic process and translation of nascent proteins in ER or membrane (Figure 5B, FDR < 0.001). These genes are putative structural constituents of the cytosolic ribosome in term of cellular components (Figure 5C, FDR < 0.001). When it comes to the term of molecular functions, they are involved in the structural constituent of ribosome, molecule activity, and RNA binding (Figure 5D, *p* < 0.01). Nine pathways related to the altered gene clusters were defined by KEGG analysis (Figure 5E, *p* < 0.05), which intriguingly were implicated in different key pathological processes such as ribosome (FDR < 0.01), metabolism (*p* = 0.014), and calcium signaling (*p* = 0.016). In view of the pathways involved (Figure 5B–E and Figure S3), we presumed that *HIVEP3* possessed essential biological functions in AML development.

### 3.6 A proposal of *HIVEP3* ‐based prognosis prediction model

**FIGURE 6:**
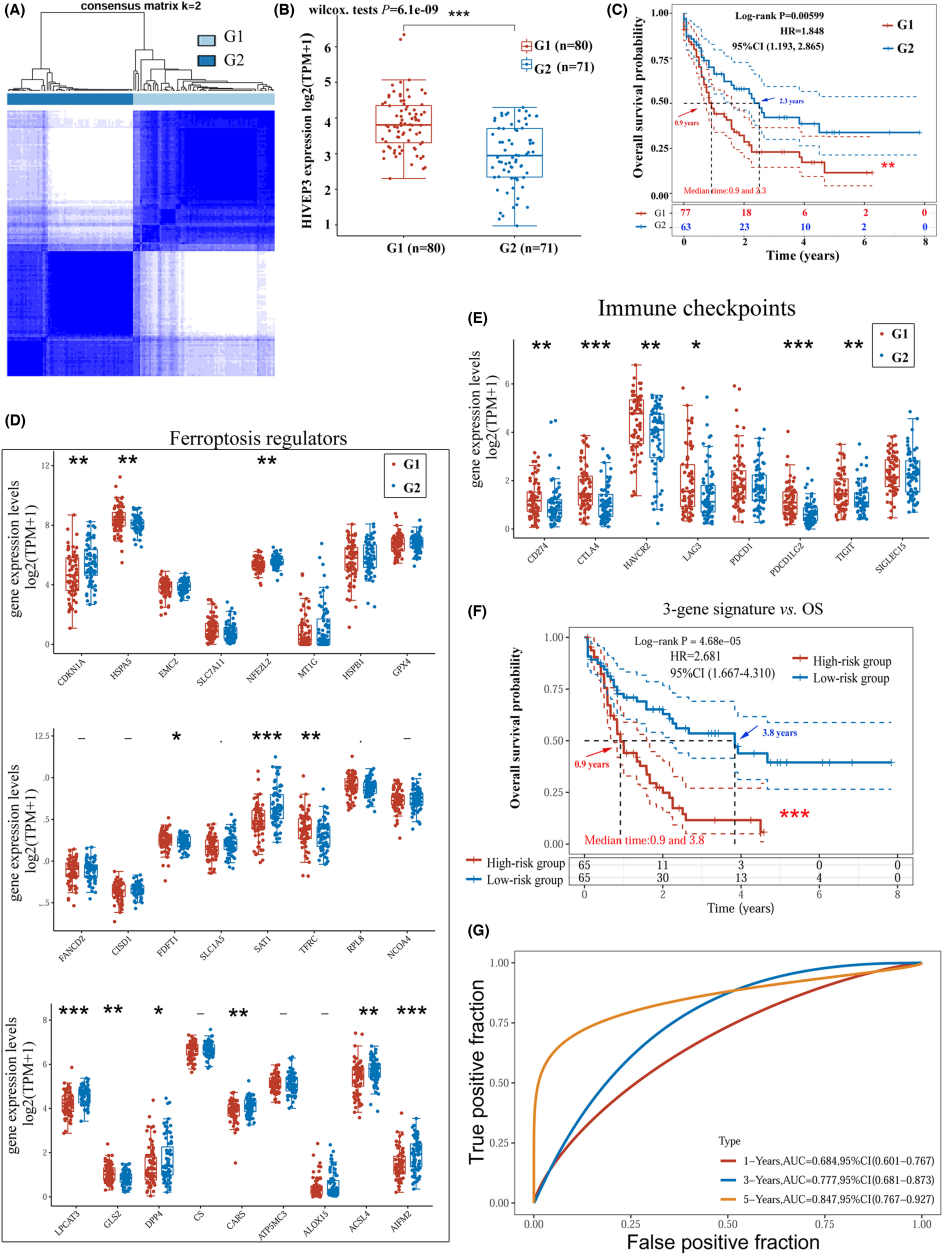
Construction and assessment of the *HIVEP3*‐cored prognostic gene model by the LASSO regression. (A) The TCGA‐LAML cohort was clustered into G1 and G2 with a consensus clustering matrix for k = 2. (B) *HIVEP3* expression levels in the two clusters (G1/G2). (C) Kaplan–Meier curves of OS for patients in G1/G2. Expression patterns of representative (D) ferroptosis regulators and (E) immune checkpoints in G1/G2. (F) Survival analysis of the three‐gene signature designated by LASSO. (G) Time‐dependent ROC analysis of the three‐gene signature for the 1‐, 3‐, 5‐year time points. The TCGA‐LAML cohort was grouped into a high‐risk score group and a low‐risk score group based on the risk score equation: riskscore = (0.0292) × *HIVEP3* + (0.1576) × *LPCAT3* + (0.1845) × *AIFM2*. Red and blue arrows indicate the median survival time

The consensus clustering analysis was supposed to help further elucidate *HIVEP3* associated pathological processes that might underlie tumorigenesis and poor outcomes (Figure 6 and Figure S4–S5). We adopted typical gene signatures from intrinsic carcinogenic pathways of tumors such as ferroptosis, immune microenvironment, and energy metabolism,^20^, ^21^, ^22^, ^23^ to divide AML patients into subgroups via consensus clustering tool (Figure S5). Expression patterns of *HIVEP3* and OS were analyzed. As it is shown in Figure S5, *HIVEP3* aberration is associated with the immune microenvironment and ferroptosis. AML subgroups with *HIVEP3* overexpression have an unfavorable prognosis.

The 200 co‐expressed genes of *HIVEP3* were synchronously subjected to consensus clustering analysis. Clustering stability was optimal at k value = 2 by the ambiguous clustering measures (Figure 6A). AML patients were divided into two subgroups: G1 and G2. G1 with *HIVEP3* overexpression has an unfavorable prognosis (Figure 6B,C). Significantly associated ferroptosis‐regulated gene signatures and immune checkpoints were filtered out. As it is shown in Figure 6D,E, *HIVEP3* aberration is associated with 18 molecules. We adopted these signatures in the LASSO regression algorithm to build a risk prediction model as below: Riskscore = (0.0292) × *HIVEP3* + (0.1576) × *LPCAT3* + (0.1845) × *AIFM2* (Figure S4C–E). The median of the risk scores was employed as the cutoff value for grouping the AML cohort into a high‐ and a low‐risk score group. KM curves indicated that the condensed three‐gene signature was associated with adverse OS (*p* = 0.000163) with an HR = 2.681 (Figure 6F). Time‐dependent ROC analysis demonstrated that the three‐gene model had an obviously larger AUC than *HIVEP3*, especially the 3‐year AUC of 0.777, and the 5‐year AUC of 0.847 (Figure 6G). *AIFM2* and *LPCAT3*, selected by the LASSO regression to assist with the prognostic evaluation of *HIVEP3*, are two essential regulators of ferroptosis. The results above implied the involvement of *HIVEP3* in the ferroptosis signaling pathway of AML tumorigenesis.

### 3.7 *HIVEP3* could be involved in the ferroptosis pathway in AML tumorigenesis

**FIGURE 7:**
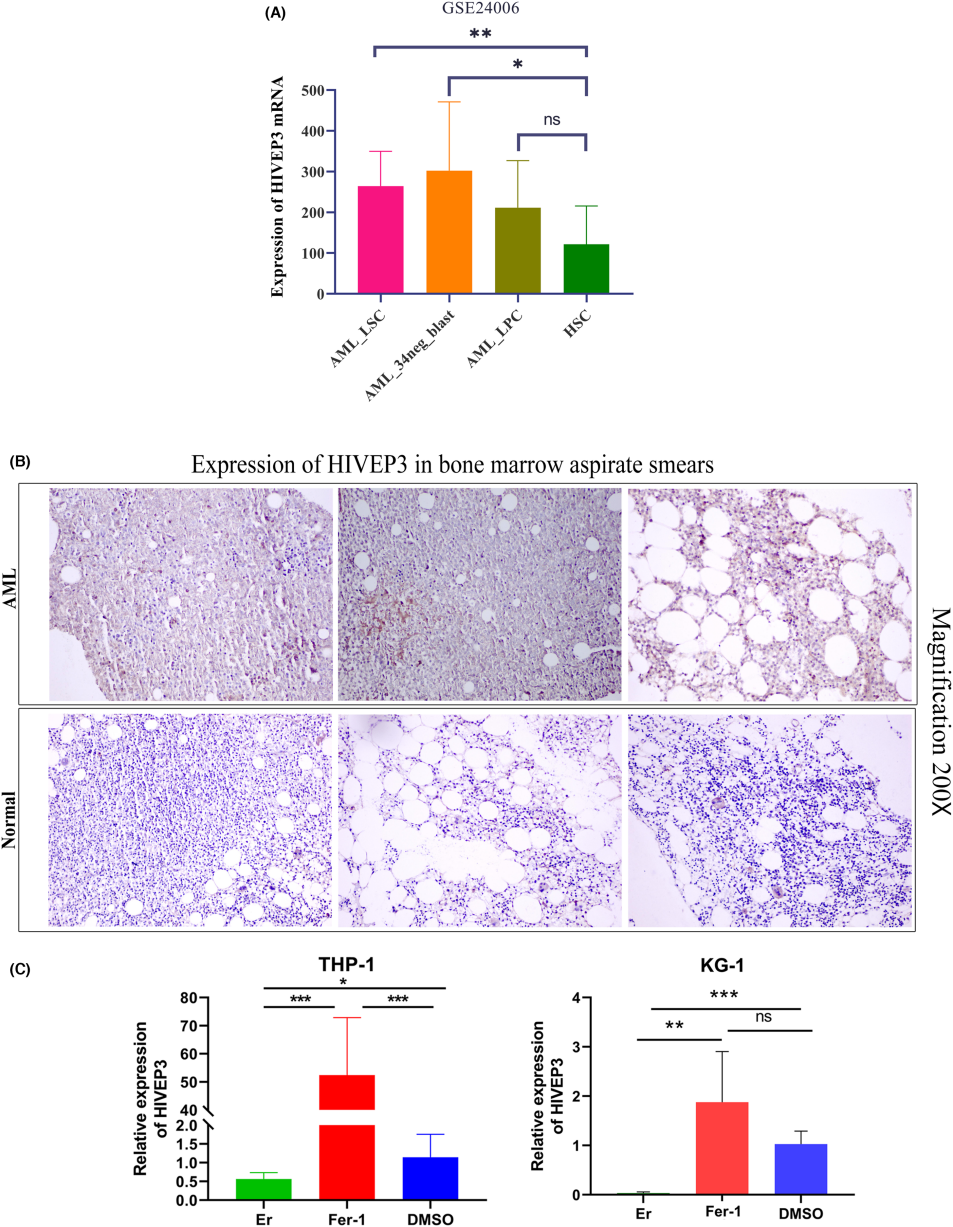
Confirmation of *HIVEP3* expression in AML patients and its involvement in ferroptosis signaling pathway. (A) *HIVEP3* mRNA expression levels in sorted leukemia cells and normal HSC from GSE24006. (B) *HIVEP3* staining in bone marrow aspirate smears of three representative AML patients and three healthy donors. (C) *HIVEP3* mRNA levels in AML cell lines THP‐1 and KG‐1 treated with ferroptosis activator 50 μM Erastin or inhibitor 1 μM ferrostatin‐1. LSC, leukemia stem cells. LPC, leukemia progenitor cells. HSC, hematopoietic stem cells. Er, Erastin. Fer‐1, ferrostatin‐1. *p < 0.05, **p < 0.01, ***p < 0.001. ns, not significant

*HIVEP3* expression discrepancy needs to be confirmed considering that the normal marrows should contain mainly mature cells, while leukemia marrows are full of immature cells. We adopted a specific dataset GSE24006, including sorted leukemic cells and normal stem cells to compare the expression level of *HIVEP3* among AML subpopulations and normal CD34^+^ HSC. *HIVEP3* expression was remarkably elevated in CD34^+^CD38^−^ AML LSC and CD34^−^ AML blast cells (Figure 7A). We further conducted an IHC assay in bone marrow aspirate smears. Cytoplasmic staining of *HIVEP3* emerged in blast cells from AML patients while positive staining was barely observed in normal bone marrow samples (Figure 7B). It determined that *HIVEP3* expression was altered at translational levels beyond the transcriptional levels. To validate the biological function of *HIVEP3* derived from bioinformatics analyses, we performed in vitro experiments. Two leukemia cell lines were treated with ferroptosis activator Erastin or inhibitor ferrostatin‐1. QRT‐PCR data displayed that *HIVEP3* expression was strongly induced when ferroptosis was inhibited. On the contrary, *HIVEP3* expression was deleted by the ferroptosis inducer (Figure 7C). It gave clues of cross‐talk between *HIVEP3* and ferroptosis signaling pathways in AML tumorigenesis.

## 4 DISCUSSION

Researchers have made many endeavors to explore significant biomarkers and prognostic indicators for AML patients and compensate for the deficits of current risk and prognostic strategy of AML. Some single genes or specific models with a group of genes were reported in the previous literatures.^3^, ^4^, ^26^, ^27^, ^28^ It is still necessary to unearth more relevant biomarkers due to the complex genetic background and mechanism of leukemogenesis. We have screened out 754 single genes with Cox‐*p* value less than 0.001 by Cox regression, such as *RHOBTB2*,^14^ *HIVEP3*, and *PSMB8*. In this study, we go further to explore the expression profiles and prognostic implications of *HIVEP* genes utilizing versatile bioinformatics tools and in vitro experiments.

We witnessed altered *HIVEP* expression in AML patients. The transcriptional levels of *HIVEP2*/*3* were remarkably elevated in AML patients compared to healthy donors and displayed the broadest range of median expression levels among pan‐cancer types. We also validated the enhanced *HIVEP3 expression* by IHC in bone marrow smears from AML patients. The unique expression patterns of *HIVEP2*/*3* in AML patients prompted us to explore whether and how it could assist with the prediction of prognosis.

The prognostic implications of *HIVEP* genes in AML were inspected and validated by GEPIA2, PrognoScan, and R studio in two separate AML cohorts. *HIVEP3* overexpression was associated with dismal outcomes in patients with AML. Based on a set of prognosis‐related variables collected in our previous study,^14^ Cox regression analyses were orchestrated subsequently to demonstrate whether *HIVEP3* could confer dismal outcomes independently. Combined with all of those prognostic variables, represented by age, leukocyte count, recurring cytogenetic abnormalities, and prognostic indicators in the Cox model,^3^, ^4^, ^14^ *HIVEP3* was manifested to retain the independence and specialty of the prognostic implications.

The current risk stratification strategy of AML patients consists of clinicopathologic characteristics, chromosomal abnormalities, and genetic alterations. The relationship between *HIVEP3* aberration and AML subgroups was examined by R studio and UALCAN to clarify whether it could contribute to risk assessment. *HIVEP3* expression was augmented in the elder age groups, non‐APL subtypes, subgroups with poor or complex cytogenetic abnormalities, and patients without *PML‐RAR* fusion or *IDH*‐R132 mutation, which coincided with the worse outcomes based on risk stratification. M0, M4, and M5 are featured by LSCs with minimal differentiation, which share the self‐renewal feature with normal HSCs. A high expression level of *HIVEP3* was confirmed in sorted leukemic cells, CD34^+^LSCs, and blast cells, but not in CD34^+^HSCs. We also recognized a notable positive association between *HIVEP3* overexpression and LSCs‐related genes such as *CD34*, *FAM30A*, and *ADGRG1* (Figure S3A).^29^ The altered *HIVEP3* transcriptional level within LSCs stemness circuits may provide a therapeutic chance. Moreover, the *HIVEP3* upregulation was parallel with the upregulation of *FLT3*, *HIF1A*, *SMAD1*, *FHL1*, and *RUNX1/3* and the downregulation of *MPO* and *VEGF*. These data reinforced the notion that *HIVEP3* could be a risk assessment tool for specific AML groups and display reciprocal relationships with the current risk stratification strategy.

Co‐expressed gene clusters of *HIVEP3* were enriched in meaningful biological categories and signaling pathways referring to ribosome, metabolism, and calcium signaling in AML. The accelerated growth and proliferation of tumor cells requires more ribosome synthesis mechanism than somatic cells.^30^ We infer that *HIVEP3*, by collaborating with the ribosomal protein (RP) family, such as *RPL15*, *RPL34*, and *RPS24*, could regulate tumor cell cycle and apoptosis and promote tumor proliferation and infiltration metastasis, angiogenesis, or other malignant biological behaviors. Calcium signaling is ubiquitous in MAPK, Wnt, JAK pathways, mediating a wide range of physiological processes. Concordantly, *HIVEP3* positively correlated with the downstream factor *TCF4* and the terminal member *SMAD3* of the Wnt signaling cascades,^31^, ^32^ *TAB2*, and *MEF2C* at the start and downstream of MAPK cascades,^33^, ^34^ and *JAK1* and *CD25* (*IL2RA*) in the JAK/STAT‐IL2 cascades^35^ (Figure S3B–D).

Previous literature has addressed the cross‐talk among tumor‐associated signaling pathways, such as ferroptosis, immune microenvironment, and metabolism. Researchers have established models based on ferroptosis‐related genes, m6A‐related genes, or immune checkpoints to predict the prognosis of AML patients.^26^, ^27^, ^28^ Nevertheless, they did not take the interplay of different signaling pathways into consideration. To further warrant the oncogenic potential of *HIVEP3*, we identified a series of immune checkpoints and ferroptosis‐related genes associated with *HIVEP3* in AML patients via consensus clustering analyses based on the expression pattern of molecules involved in ferroptosis, immune microenvironment, and hypoxia. An efficient LASSO model was proposed to enhance the predictive accuracy of *HIVEP3* with the assistance of ferroptosis regulators *AIFM2* and *LPCAT3*. *LPCAT3* (Lysophospholipid acyltransferase 3) promotes the incorporation of PUFAs into phospholipids to form PUFA‐containing phospholipids (‐PLs), which are substrates for pro‐ferroptotic lipid peroxidation.^36^ Knockdown or knockout of *LPCAT3* may suppress ferroptosis triggered by RSL3 and Erastin. The *AIFM2*, an NADH oxidase also known as *FSP1*, which has a context‐dependent role in protecting against oxidative damage by using CoQ10 as a substrate, can prevent ferroptosis.^37^ We treated AML cells in vitro with compounds Erastin or ferrostatin‐1 to mimic the role of ferroptosis regulators. *HIVEP3* expression level could be induced by Fer‐1, while depleted by Er. Given the versatile roles of *HIVEP3* in apoptosis and immune response, the existence of synergistic or complementary effects between ferroptosis and *HIVEP3*‐mediated pathways is possible. Although the LASSO technique could be optimistic, it would be hard for physicians to use because the collection of NGS data and the preprocessing algorithm needs professional bioinformatics and computer skills.^38^ We have yet to confirm the applicability of the LASSO model in additional data sets from the public or clinical source. Herein, we reported it as a rationalistic model so far. Due to its limited practical value, we conducted in vitro experiments to confirm the *HIVEP3* alteration in AML cells treated with Er or Fer‐1. *HIVEP3* augmentation triggered by ferroptosis impairment made it a convincing tumorigenic marker and survival predictor. However, and importantly, considering the double‐edged sword role of ferroptosis in immune therapy and disease progression, more in‐depth experiments need to be designed to explore the exact molecular functions of *HIVEP3*.

In conclusion, for the first time, we profiled the organ‐specific expression patterns and assessed the prognostic implications of *HIVEP* genes in AML patients in this study through data mining and in vitro experiments. The fatal tumorigenic signaling pathways were taken into consideration to get a complete understanding of the biological roles. Our results illustrated that *HIVEP3* was intriguingly elevated in AML, particularly in high‐risk subgroups, making it a promising biomarker. Not only could *HIVEP3* itself be applied as an independent prognostic indicator in clinical practice, it could also cooperate with ferroptosis regulators to confer adverse outcomes for AML patients through a computed LASSO model. Therefore, *HIVEP3* was expected to be utilized by physicians as a marker when they choose the opportunity and timing of usage of ferroptosis regents and immune therapy. We await further in‐depth laboratory work of this discovery to improve the predictive accuracy of prognosis and interpret the intricate cross‐talk of signaling pathways related to leukemogenesis.

## AUTHOR CONTRIBUTION

WSM and XNZ conceptualized and designed the study. XYZ organized databases. PL performed the statistical analyses under R studio. KL validated the statistical analyses. WWL and JZW drafted the first manuscript. XNZ arranged figures and acquired the funding. All authors contributed to manuscript revision, read, and approved the submitted version.

## CONFLICT OF INTEREST

The authors have no conflict of interest.

## ETHICS STATEMENT

This research was done with the approval of the Medical Ethics Committee of Shandong First Medical University according to the Declaration of Helsinki.

## Supporting information


Data S1

Table S1

Figure S1–S5

## Data Availability

The datasets analyzed for this study can be found in the National Cancer Institute (NCI) TCGA cancers (TCGA‐LAML) https://portal.gdc.cancer.gov/, GTEx (normal tissues) https://gtexportal.org/home/datasets, and Gene Expression Omnibus (GEO: GSE12417, GSE24006) https://www.ncbi.nlm.nih.gov/geo. All data that support the findings of this study are available from the corresponding author upon reasonable request.
