# Supplementary material for: HIVEP3 cooperates with ferroptosis gene signatures to confer adverse prognosis in acute myeloid leukemia
Source: Cancer Med. 2022 May 10;11(24):5050–65. doi: 10.1002/cam4.4806 (PMC9761064; doi:10.1002/cam4.4806)
Supplement: Supplementary file 1 — Data S1 [file CAM4-11-5050-s001.docx]

# Supplementary information

**Databases**

**GEPIA2** (Gene Expression Profiling Interactive Analysis 2, <http://gepia2.cancer-pku.cn/>, Beijing, China) has a collection of cancer-related RNA sequencing expression data derived from TCGA and the GTEx projects. Survival analysis was conducted through the “Expression Analysis” module in the TCGA-LAML cohort (n=151), survival status, and RNA-seq data of which are reposited in the “dataset sources” panel. Cases with incomplete clinical data were omitted.

**PrognoScan database** (<http://gibk21.bse.kyutech.ac.jp/PrognoScan/index.html>) enables systematic meta-analysis for the prognostic value of gene candidates employing the minimum *P*-value approach for grouping patients based on continuous gene expression measurement. We performed the prognosis analysis for the *HIVEP3* in a normal karyotype AML CG (1999-2003) cohort (n=163, GSE12417-GPL97 dataset, <https://www.ncbi.nlm.nih.gov/geo/query/acc.cgi?acc=GSE12417>). The cox regression results were plotted as Kaplan-Meier curves with cox coefficient and *P* values from a log-rank test. The *P*-value cutoff was set at 0.05.

**LinkedOmics** (<http://www.linkedomics.org>) provides a unique platform to analyze and visualize cancer multi-omics data and clinical data of TCGA tumor samples. Associated genes with *HIVEP3* were defined, presented in volcano plots in the TCGA-LAML cohort (n=173). The Pearson Correlation test was adopted to evaluate the relationship between an individual attribute of interest and a large attribute pool.

**UALCAN** (Birmingham, AL, USA, <http://ualcan.path.uab.edu>) facilitates the comprehensive variations of specific genes and survival associations across TCGA cancer types. *HIVEP3* expression in AML sub-groups with specific clinicopathologic features was unearthed through the “Expression Analysis” module. The level3 RNA-seq data and survival profiles of the AML cohort (n=163) are publicly available via TCGA assembler (<http://www.compgenome.org/TCGA-Assembler/>). The student’s t-test was used to perform expression analysis. A *P*-value=0.05 was considered as the threshold of the statistical significance.

**The Database WebGestalt** (<http://www.webgestalt.org/option.php>) for translating gene lists into biological insight was utilized to conduct Gene Ontology (GO) and Kyoto Encyclopedia of Genes and Genomes (KEGG) pathway enrichment analysis for *HIVEP3* and co-expressed gene set. The built-in reference set of human protein-coding genome was selected as the background parameter. Representative functional categories are annotated in the color gradient bubble charts drawn by R packages “ggplot2” and “clusterProfiler”. In the enrichment result, ( -log10) *P*-value＞1.3 is considered to be enriched to a meaningful pathway.

**Availability of datasets**

The datasets analyzed for this study can be found in the National Cancer Institute (NCI) TCGA cancers (TCGA-LAML) <https://portal.gdc.cancer.gov/>, GTEx (normal tissues) <https://gtexportal.org/home/datasets>, and Gene Expression Omnibus (GEO: GSE12417) <https://www.ncbi.nlm.nih.gov/geo>.

**Table S**1**.** Representative co-expressed genes of *HIVEP3* from LinkedOmics and survial analysis in the TCGA-LAML cohort.

**Supplementary Figure legends**

**Fig. S1.** Univariate Cox regression analyses of common clinical features and *HIVEP3* in the TCGA-LAML cohort via R studio. The forest plots were drawn with the *P*-values, HR, and 95%CI of each variable through ‘forestplot’ R package. The cutoff value was set at nominal *P*<0.05. ******P*＜0.05, *******P*＜0.01, ********P*＜0.001.

**Fig. S2.** The correlations between *HIVEP3* and disease-related genes in the TCGA-LAML cohort (GEPIA2). The scatter plots show the correlation between HIVEP3 and disease-related genes such as *FLT3*, *HIF1A*, *SMAD1*, *FHL1*, *RUNX1/3*, *MPO*, and *VEGF* according to Pearson’s correlation analysis (GEPIA2). A non-log scale of mRNA expression levels was used for calculation and a log2-scale axis was used for visualization. R values present correlation coefficients. A *P*-value < 0.05 was considered statistically significant.

**Fig. S3.** Association of *HIVEP3* and genes involved in LSCs features and vital signaling pathways in AML by Pearson’s correlation analyses. Correlation between *HIVEP3* and (A) LSCs-related genes including *FAM30A*, *ADGRG1*, *CD34*, *ZBTB46*, and *NYNRIN*, (B) *MEF2C* and *TAB2* involved in MAPK pathways, (C) *IL2RA* and *JAK1* in JAK/STAT pathways and (D) *SMAD3* and *TCF4* in Wnt pathways. R values indicate correlation coefficients. The cutoff value was set at *P*-value < 0.05.

**Fig. S4.** Consensus clustering analysis and the LASSO model. (A) Cumulative distribution function (CDF) for k=2 to 6 in consensus clustering analysis by *HIVEP3* and co-expressed genes in the TCGA-LAML cohort. (B) Relative change in area under the CDF curve for k=2 to 6. (C) LASSO coefficient profiles of the 17-gene set including *HIVEP3*, ferroptosis regulators (*LPCAT3, AIFM2, CDKN1A, HSPA5, NFE2L2, FDFT1, SAT1, TFRC, GLS2, CARS, ACSL4*) and immune checkpoints (*CD274, HAVCR2, CTLA4, LAG3, PDCD1LG2,* and *TIGIT*) in the TCGA-LAML cohort. (D) Partial likelihood deviance for tuning the parameter selection in the LASSO regression model. (E) AML patients were divided into the high- and low-risk scores subgroups with the median risk score as cutoff value (grey dotted lines). Risk scores were calculated by a LASSO equation: riskscore=(0.0292)×*HIVEP3*+(0.1576) ×*LPCAT3*+(0.1845) ×*AIFM2*. Survival status and gene expression levels of *HIVEP3*, *LPCAT3*, and *AIFM2* of each patient were displayed in the panels below.

**Fig. S5.** AML patients in the TCGA-LAML cohort were clustered by consensus clustering analyses based on (A) immune checkpoints, (B) ferroptosis regulators, (C) DNA damage regulators, (D) hypoxia regulators, (E) m6A regulators. *HIVEP3* expression levels were compared between two subgroups, respectively (the middle panels). KM curves showed the overall survival probability (the right panels). A *P*-value < 0.05 defined statistical significance. ******P*＜0.05, *******P*＜0.01, ********P*＜0.001, ns, not significant.
