# Supplementary material for: HIVEP3 cooperates with ferroptosis gene signatures to confer adverse prognosis in acute myeloid leukemia
Source: Cancer Med. 2022 May 10;11(24):5050–65. doi: 10.1002/cam4.4806 (PMC9761064; doi:10.1002/cam4.4806)

Figure S1.

Univariate cox regression in the TCGA-LAML cohort (n=151)

Characteristics	HR (95% CI)	P value
Age (≥60 years old)	2.8 (1.7-4.4)	<0.001 ***
Leukocyte	1.052 (0.718-1.542)	0.018 *
blast cell percentage_ (bone marrow)	1.003 (0.995-1.011)	0.232
blast cell percentage_ (peripheral blood)	1.001 (0.995-1.007)	0.537
cytogenetic risk		
intermediate/normal	3.7(1.6-8.5)	0.002 **
poor	3.1(1.5-6.5)	0.003 **
FLT3_mutation	1.9 (1.2-3.1)	0.009 **
DNMT3A_mutation	2.6 (1.4-4.4)	<0.001 ***
RUNX1_mutation	1.6 (0.86-3.1)	0.135
NPM1_mutation	1.3 (0.82-2.2)	0.234
IDH2_mutation	1.0 (0.53-2)	0.918
IDH1_mutation	0.89 (0.43-1.9)	0.765
TET2_mutation	1.8 (0.87-3.5)	0.548
TP53_mutation	5.9 (2.6-13)	<0.001 ***
NRAS_mutation	0.88 (0.28-2.8)	0.831
CEBPAdouble_mutation	1.3 (0.6-2.6)	0.551
WT1_mutation	1.0 (0.44-2.4)	0.954
PTPN11_mutation	1.1 (0.45-2.8)	0.807
KRAS_mutation	1.1 (0.45-2.8)	0.815
HIVEP3_high	1.4(1.1-1.8)	0.003 **
HIVEP1_high	1.1(0.76-1.6)	0.582
HIVEP2_high	0.84(0.69-1.0)	0.094

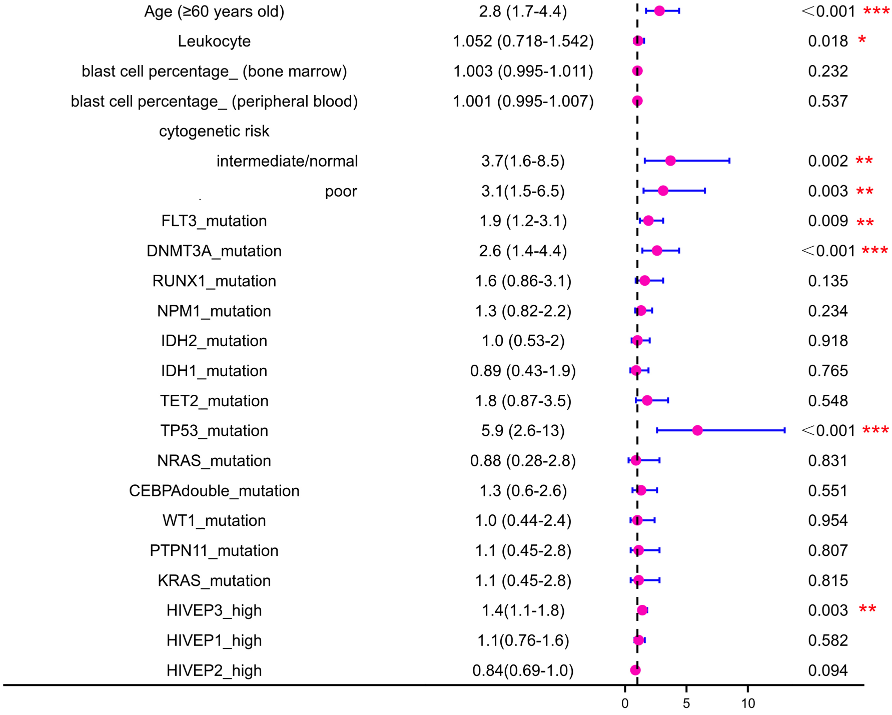

Figure S1.

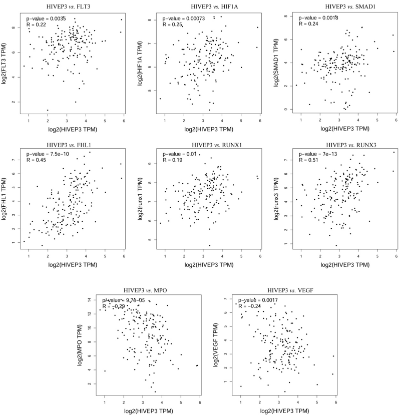
HIVEP3 *vs.* FLT3
p−value = 0.0035
R = 0.22
log2(FLT3 TPM)
log2(HIVEP3 TPM)
HIVEP3 *vs.* HIF1A
p−value = 0.00073
R = 0.25
log2(HIF1A TPM)
log2(HIVEP3 TPM)
HIVEP3 *vs.* SMAD1
p−value = 0.0018
R = 0.24
log2(SMAD1 TPM)
log2(HIVEP3 TPM)
HIVEP3 *vs.* FHL1
p−value = 7.5e−10
R = 0.45
log2(FHL1 TPM)
log2(HIVEP3 TPM)
HIVEP3 *vs.* RUNX1
p−value = 0.01
R = 0.19
log2(runx1 TPM)
log2(HIVEP3 TPM)
HIVEP3 *vs.* RUNX3
p−value = 7e−13
R = 0.51
log2(runx3 TPM)
log2(HIVEP3 TPM)
HIVEP3 *vs.* MPO
p−value = 9.7e−05
R = −0.29
log2(MPO TPM)
log2(HIVEP3 TPM)
HIVEP3 *vs.* VEGF
p−value = 0.0017
R = −0.24
log2(VEGF TPM)
log2(HIVEP3 TPM)

Figure S3.

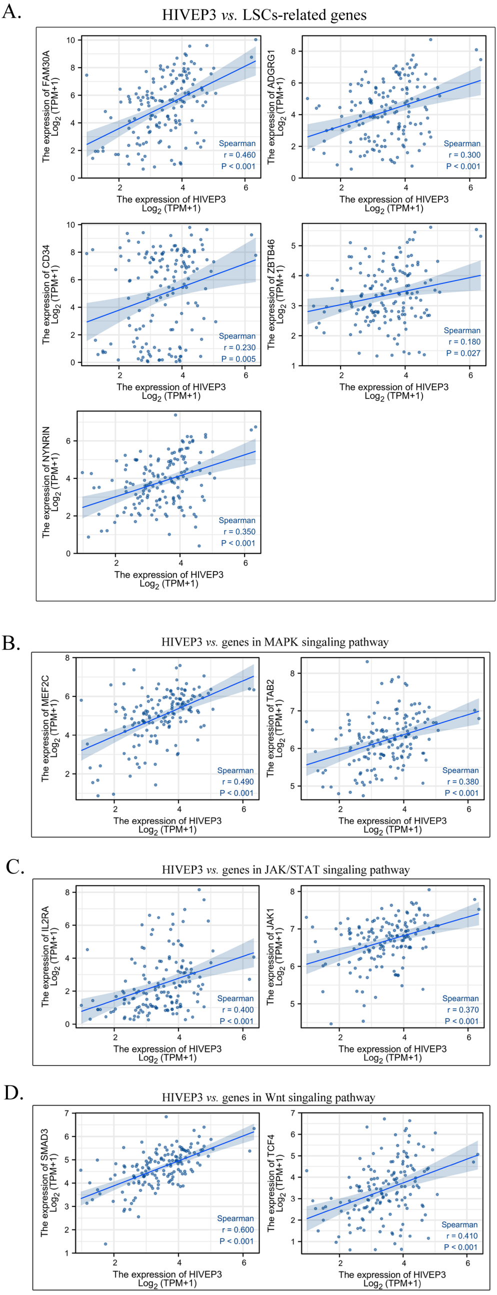

A.
HIVEP3 vs. LSCs-related genes
The expression of FAM30A
Log2 (TPM+1)
Spearman
r = 0.460
P < 0.001
The expression of ADGRG1
Log2 (TPM+1)
Spearman
r = 0.300
P < 0.001
The expression of CD34
Log2 (TPM+1)
Spearman
r = 0.230
P = 0.005
The expression of ZBTB46
Log2 (TPM+1)
Spearman
r = 0.180
P = 0.027
The expression of NYNRIN
Log2 (TPM+1)
Spearman
r = 0.350
P < 0.001
The expression of HIVEP3
Log2 (TPM+1)
B.
HIVEP3 vs. genes in MAPK singaling pathway
The expression of MEF2C
Log2 (TPM+1)
Spearman
r = 0.490
P < 0.001
The expression of TAB2
Log2 (TPM+1)
Spearman
r = 0.380
P < 0.001
The expression of HIVEP3
Log2 (TPM+1)
C.
HIVEP3 vs. genes in JAK/STAT singaling pathway
The expression of IL2RA
Log2 (TPM+1)
Spearman
r = 0.400
P < 0.001
The expression of JAK1
Log2 (TPM+1)
Spearman
r = 0.370
P < 0.001
The expression of HIVEP3
Log2 (TPM+1)
D.
HIVEP3 vs. genes in Wnt singaling pathway
The expression of SMAD3
Log2 (TPM+1)
Spearman
r = 0.600
P < 0.001
The expression of TCF4
Log2 (TPM+1)
Spearman
r = 0.410
P < 0.001
The expression of HIVEP3
Log2 (TPM+1)

Figure S4.

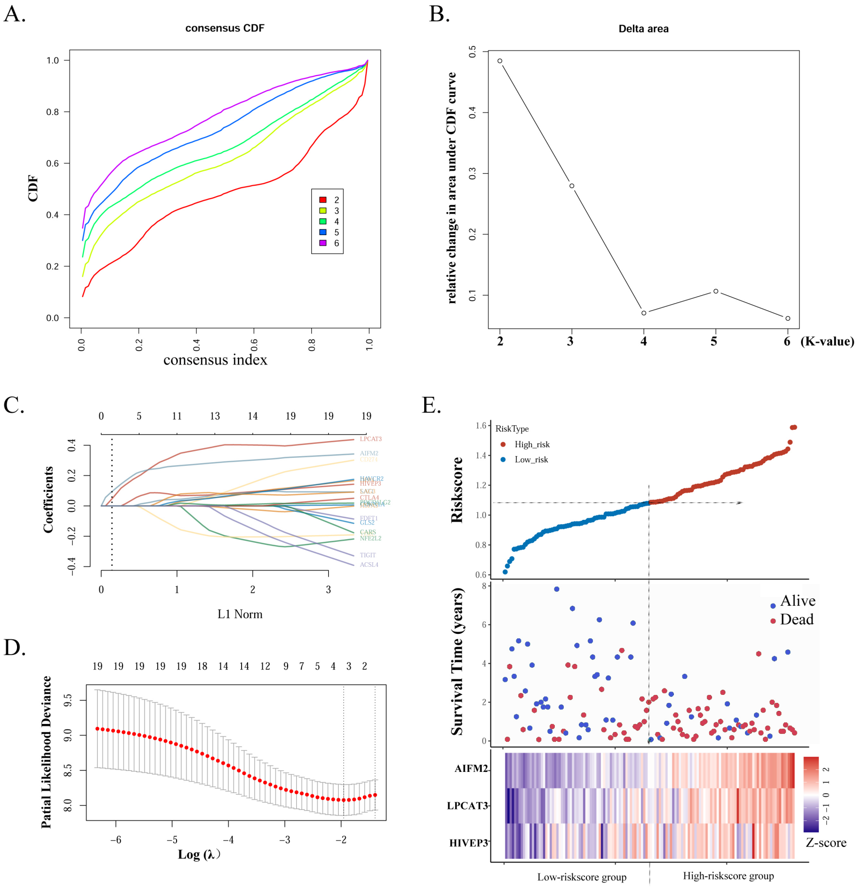
A.
consensus CDF
CDF
consensus index
B.
Delta area
relative change in area under CDF curve
(K-value)
C.
Coefficients
L1 Norm
LPCAT3
AIFM2
HAVCR2
HIVEP3
CTLA4
CARS
NFE2L2
TIGIT
ACSL4
D.
Patial Likelihood Deviance
Log (λ)
E.
RiskType
High_risk
Low_risk
Riskscore
Survival Time (years)
Alive
Dead
AIFM2
LPCAT3
HIVEP3
Z-score
Low-riskscore group
High-riskscore group

Figure S5.

A.

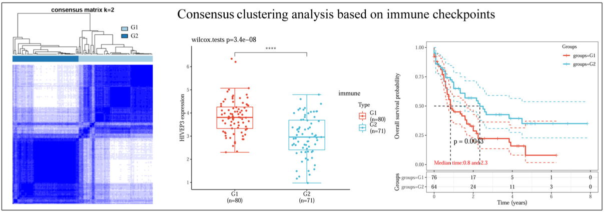

B.

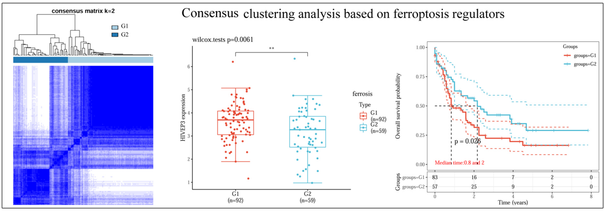

C.

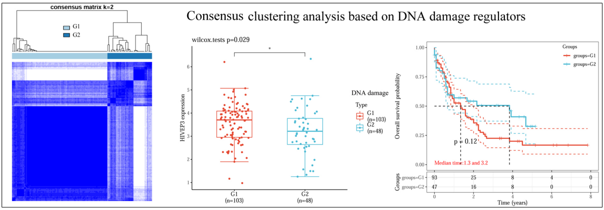

D.

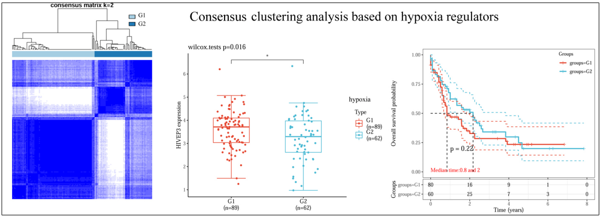

E.

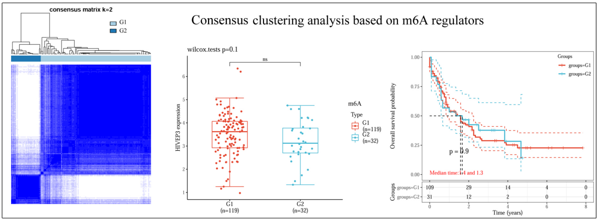

Supplement: Supplementary file 3 — Figure S1–S5 [file CAM4-11-5050-s003.pdf]
